# Extracellular vesicle microRNA quantification from plasma using an integrated microfluidic device

**DOI:** 10.1038/s42003-019-0435-1

**Published:** 2019-05-20

**Authors:** Zeinab Ramshani, Chenguang Zhang, Katherine Richards, Lulu Chen, Geyang Xu, Bangyan L. Stiles, Reginald Hill, Satyajyoti Senapati, David B. Go, Hsueh-Chia Chang

**Affiliations:** 1 Department of Chemical and Biomolecular Engineering, University of Notre Dame, Notre Dame, IN 46556 USA; 2 Center for Microfluidics and Medical Diagnostics, University of Notre Dame, Notre Dame, IN 46556 USA; 3 Harper Cancer Research Institute, University of Notre Dame, Notre Dame, IN 46556 USA; 4 Department of Biological Sciences, University of Notre Dame, Notre Dame, IN 46556 USA; 5 Pharmacology & Pharmaceutical Sciences, School of Pharmacy, University of Southern California, Los Angeles, CA 90211 USA; 6 Department of Physiology, School of Medicine, Jinan University, Guangzhou, 510632 Guangdong China; 7 Lawrence J. Ellison Institute for Transformative Medicine of USC, University of Southern California, Beverly Hills, CA 90211 USA; 8 Keck School of Medicine, University of Southern California, Los Angeles, CA 90033 USA; 9 Department of Aerospace and Mechanical Engineering, University of Notre Dame, Notre Dame, IN 46556 USA

**Keywords:** Lab-on-a-chip, RNA probes, miRNAs

## Abstract

Extracellular vesicles (EV) containing microRNAs (miRNAs) have tremendous potential as biomarkers for the early detection of disease. Here, we present a simple and rapid PCR-free integrated microfluidics platform capable of absolute quantification (<10% uncertainty) of both free-floating miRNAs and EV-miRNAs in plasma with 1 pM detection sensitivity. The assay time is only 30 minutes as opposed to 13 h and requires only ~20 μL of sample as oppose to 1 mL for conventional RT-qPCR techniques. The platform integrates a surface acoustic wave (SAW) EV lysing microfluidic chip with a concentration and sensing microfluidic chip incorporating an electrokinetic membrane sensor that is based on non-equilibrium ionic currents. Unlike conventional RT-qPCR methods, this technology does not require EV extraction, RNA purification, reverse transcription, or amplification. This platform can be easily extended for other RNA and DNA targets of interest, thus providing a viable screening tool for early disease diagnosis, prognosis, and monitoring of therapeutic response.

## Abstract (web summary)

Zeinab Ramashani, Chenguang Zhang et al. developed a microfluidic device that allows for quantification of extracellular vesicle (EV) microRNA from plasma. The platform does not require EV extraction, so it is able to process samples in as little as 30 minutes and requires much smaller volumes of sample than competing platforms.

## Introduction

MicroRNAs (miRNAs) are small strands (~22 nucleotides) of noncoding RNAs that function as posttranscriptional gene regulators by binding to their target messenger RNAs. miRNAs are transported between cells by small extracellular vesicles (EVs), secreted membrane vesicles that range in size from ~30 to 150 nm and overall are less than 200 nm in diameter^1–3^. These EVs are secreted by most cells and abundant in many bodily fluids such as blood, saliva, urine, and breast milk^4^. Further, miRNAs contained inside EVs have been postulated to play a key role in cancer proliferation^5,6^. Consequently, EVs and the miRNA they carry (EV-miRNAs) are promising circulating biomarkers candidates for early-stage disease diagnosis^7^.

However, conventional methods of EV-miRNA analysis and quantification are plagued by inefficiencies. Depending on the sample source (e.g., whole blood), the process involves multiple steps of purification, followed by EV isolation, using antibody capture^8^, ultracentrifugation^7^, or polymeric precipitation (e.g., the commercial ExoQuick^®^ kit)^9^. The collected EVs are then chemically lysed, and the released miRNAs are purified for downstream RT-qPCR analysis^10^. However, due to low extraction, ligation, and amplification efficiencies, the conventional RT-qPCR procedure produces large quantification uncertainties^11^. Spike-in control miRNAs such as cel-mir-39-5p are often added during RT-qPCR for normalization^12^. Moreover, different miRNAs can have very different extraction, ligation, and amplification yields, suggesting that even normalized expression levels for different target miRNAs, cannot be quantitatively compared^13^. Until now, very few miRNA biomarkers are FDA approved for liquid biopsy^14^. In fact, even the average number of miRNAs per vesicle varies by orders of magnitude across different reports^1,15–19^.

Despite significant advances in microfluidic diagnostic platforms in recent years, there are few reports on the development of miRNA assays. Most of the reported platforms utilize chemical lysing, thus requiring rigorous sample treatment steps prior to downstream target detection using PCR or other fluorescence-based sensing techniques^20–22^. Due to multistep sample purification processes, current microfluidic systems suffer from low yield, poor sensitivity, or large detection bias. Thus while the use of small sample volumes has made microfluidic systems attractive for cancer diagnosis, a chemical-free EV lysing and label-free biomarker detection platform is needed.

In this work, we integrate chemical-free EV lysing and membrane sensor technologies into a PCR-free microfluidic diagnostic platform for one-step absolute quantification of EV-miRNAs from untreated plasma samples. The assay time is reduced by hours, the sample volume by over an order of magnitude, and quantification error/bias associated with sample transfer, EV extraction, miRNA purification, ligation, and amplification are eliminated. We verify this new method by accurate quantification of a specific target free-floating and EV-miRNA in untreated human and mouse plasma samples, validated with RT-qPCR.

## Results

### Design of the integrated microfluidic platform

**Fig. 1:**
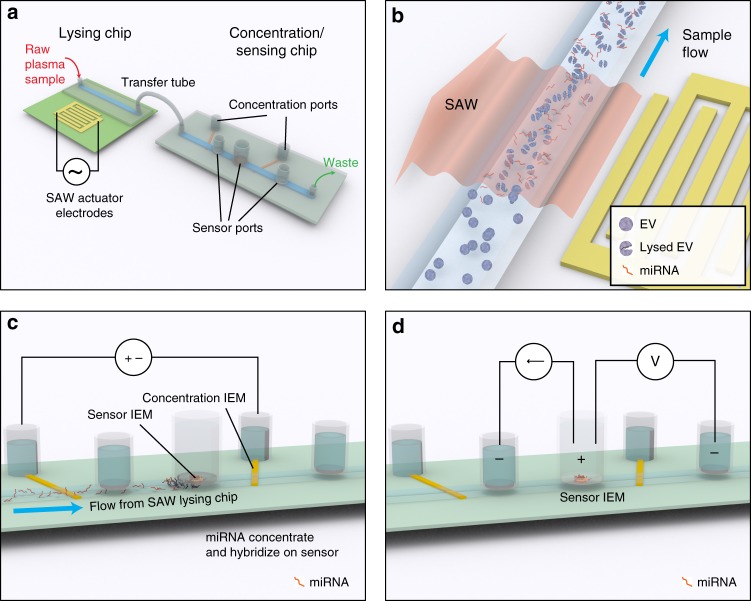
Design and operation of integrated microfluidic platform for EV-miRNA analysis. **a** The device consists of two chips, a lysing chip and concentration/sensing chip, connected by a simple transfer tube such that sample is analyzed under continuous flow. **b** The principle of lysing using SAWs, where the SAWs refract into the microchannel and generate an acoustic pressure that shears the EVs open. SAWs are induced by 20 interdigitated electrodes on the surface of a 128° YX lithium niobate (LiNbO_3_) piezoelectric substrate with a polycarbonate microchannel attached to the surface using double-sided Kapton^®^ tape. **c** The principle of IEM concentration, where a voltage is applied between two reservoirs separated by a CEM, creating an accumulation of negative space charge, including negatively charged miRNA, upstream of the CEM. **d** The principle of IEM sensing, where an AEM is functionalized with oligo probe complements of a target miRNA. After miRNA hybridization, the current–voltage relationship across the AEM sensor is shifted in the over-limiting region, and the voltage shift (Δ*V*) can be directly correlated with miRNA concentration

The microfluidic platform is designed to efficiently lyse EVs, concentrate the released miRNA for detection, and quantify a target miRNA biomarker. Figure 1a shows the overall architecture of the device, which consists of a surface acoustic wave (SAW) lysing chip that mechanically lyses the EVs and a second concentration/sensing chip where two sets of ion-exchange membranes (IEMs) are used to concentrate and quantify the target miRNA, respectively.

Surface acoustic waves are mechanical Rayleigh waves that travel along the surface of a piezoelectric crystal^23^. Formed by exciting interdigitated electrodes on the surface of the chip at radio frequencies, the SAW propagates away from the electrodes and refracts into liquid on the surface, producing an acoustic pressure that can induce both cell^24^ and exosome^25^ lysis. Here, a polycarbonate microfluidic channel is built on the surface of a 128° YX lithium niobate (LiNbO_3_) piezoelectric substrate, parallel to the interdigitated electrodes, such that the SAWs refract into the channel and lyse the EVs under continuous flow (Fig. 1b).

The external concentration polarization phenomena of IEMs and inherent negative charge of nucleic acids are used to concentrate and quantify the target miRNA. IEMs are nanoporous membranes with a high positive (anion-exchange) or negative (cation-exchange) surface charge that allow only the transport of counter ions through the membrane under an applied electric field. This counter-ion current reduces the ionic strength on one side of the membrane (known as the depletion zone) and increases the ionic strength on the other side (known as the enrichment zone)^26^. We have developed protocols to precisely control the dimensions of the depletion and enrichment zones^27^.

In order to concentrate the RNA by as much as three orders of magnitude^26^, a CEM is positioned such that the boundary of the depletion zone is directly below the sensor (Fig. 1c). For detection of target miRNA, the depletion side of an anion-exchange membrane (AEM) is used where the hybridization of target miRNA with oligoprobes attached to the AEM reduces the ion depletion action^26,28^. (It should be noted that the nanopores of the AEM only allow the passage of counter ions but not nucleic acids.) As a result, the onset voltage for electro-convection responsible for the over-limiting current in the CVC (current–voltage curve) is shifted by several volts^26,28^. This large voltage shift, due to gating of the depletion ion current by the hybridized miRNAs, is much larger than voltage signals from electrochemical sensors and offers sensitive quantification^28^ (Fig. 1d).

The IEM concentrator and sensor are built into a polycarbonate microfluidic channel on a second polycarbonate chip that takes flow directly from the lysis chip through a microtube. This enables uninterrupted sample transfer to ensure there is no sample loss between steps in the analysis and eliminates the need for purification or preparation protocol between stages.

### Device operation and sensor calibration

**Fig. 2:**
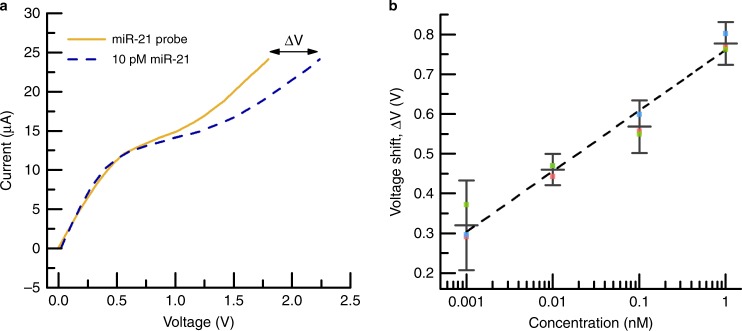
Characterization and calibration of the IEM sensor. **a** A representative set of CVCs where the current response of the IEM sensor is plotted as a function of voltage. Initially, a CVC is acquired for the IEM sensor functionalized with the miR-21 probe but only in 1× PBS (labeled ‘miR-21 probe’). A CVC is then acquired for the same sensor but in a solution of 1× PBS containing 10 pM miR-21 (labeled ‘10 pM miR-21’). Upon miR-21 hybridization with the probes attached to the IEM, a large voltage shift Δ*V* occurs in the over-limiting region, and this can be correlated to the miR-21 concentration in solution. **b** Calibration curve relating the IEM sensor voltage shift (Δ*V*) to miR-21 concentration where the error bars represent the uncertainty at 95% confidence for three replicates for each point. The dash line is a linear curve fit reflecting log-linear behavior

The integrated device operates on a specific timing strategy that has been optimized to ensure that an injected sample undergoes sufficient exposure during each step of the analysis (lysing—concentration—sensing). To initiate the analysis, ~20 μL of sample followed by 60 μL of 1× PBS (phosphate buffered saline) (as driver fluid) is injected into the microchannel on the lysing chip at 250 μL h^−1^, while SAWs are generated at ~1 W. At this flow rate, the sample resides in the microchannel and is exposed to SAWs for 1 min, estimated to be sufficient to lyse ~100% of EVs before saturation (Supplementary Fig. 1). Sample exiting the SAW lysing chip flows directly into the concentration/sensing chip through a 500 µm diameter, 10-cm-long Tygon^®^ tube. A voltage of 200 V is applied for 20 min between the concentration IEM reservoir and a reference reservoir (Fig. 1c) inducing ion depletion and subsequent miRNA concentration directly beneath the IEM sensor. This concentration step is maintained for 20 min so that the target miRNAs hybridize with their complimentary oligoprobes attached to the IEM sensor. The microfluidic channel is subsequently washed using 4× PBS to eliminate nonhybridized species from the membrane surface and 0.1× PBS is pumped into the channel prior to measurement. Finally, the concentration voltage is terminated, and a current–voltage sweep is applied between the sensing IEM reservoir and reference reservoir (Fig. 1d), producing a CVC (Fig. 2a).

We used miRNA miR-21, whose up/downregulation has been correlated with pancreatic^29^ and lung cancer^30^ among many others^31–33^, as our model biomarker. The IEM sensor was functionalized with a complementary oligoprobe to miR-21 using a well-documented standard process^28^ (see “Methods”) and calibrated by exposing it to different concentrations (1 pM to 1 nM) of commercially-purchased miR-21 in 1× PBS using the above procedure. Figure 2a shows the CVC for the IEM after oligo functionalization and after exposure to 10 pM of miR-21, where a voltage shift of Δ*V* = 0.44 V was measured at a current of 24 μA. As shown in Fig. 2b, the miR-21 concentration (*C* in nM) and voltage shift (Δ*V* in V) are exponentially related by $C = {\mathrm{exp}}\left( {\frac{{\Delta V - 0.76145}}{{0.06614}}} \right)$. The sensor exhibited log-linear behavior over three decades of concentration with a limit of detection of 1 pM. In order to benchmark the IEM sensor, a known concentration of synthetic miR-21 was diluted in 1× PBS and tested with both the integrated device and conventional RT-qPCR. Supplementary Fig. 2 shows that the calculated average expression levels for both methods are nearly identical. (See “Methods” for expression level calculation.)

### Principle of operation for analyzing plasma EV-miRNAs

Free-floating miRNA in plasma and other human fluids can be as numerous as^34^ or even exceed^35^ EV-miRNAs. However, EV-miRNAs released by diseased cells have been speculated to be the most promising biomarker for early cancer diagnosis rather than free-floating miRNAs released by dead or white blood cells^34–36^. For accurate use of miRNA as a potential biomarker for disease, it is necessary to distinguish between EV-miRNAs and free-floating miRNAs.

**Fig. 3:**
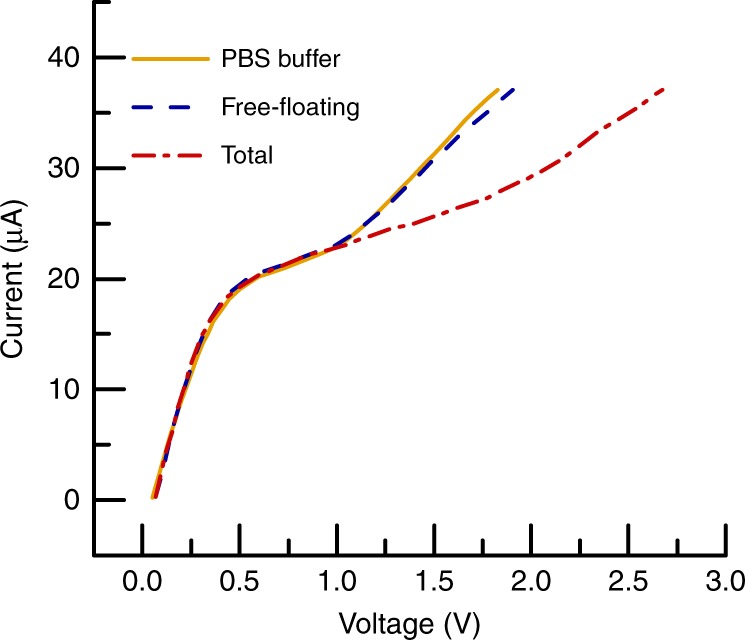
Representative CVCs for the integrated microfluidic platform using healthy human plasma illustrating the principle of operation for analyzing plasma EV-miRNA. The representative CVCs are for three analyses: the miR-21 oligoprobe on the IEM sensor in only buffer solution (PBS buffer), the unlysed plasma sample (free-floating), and the SAW-lysed plasma sample (total). The relative voltage shifts reflect the concentration of free-floating miR-21(Δ*V* ≈ 0.1 V) and total (free-floating plus EV-contained) miR-21 (Δ*V* ≈ 0.7 V) in healthy human plasma, respectively

For each plasma miRNA concentration measurement, an initial measurement is conducted with no sample passed through the device, with 0.1× PBS in the microchannel, to establish a baseline CVC for only the oligoprobes on the IEM sensor. Following this step, 20 μL of plasma sample is injected into the integrated platform while the SAW device is off, passing through the lysing chip and directly into the concentration/sensing chip with all other parts of the procedure remaning the same. The resulting CVC and corresponding voltage shift are thus due to only free-floating target miRNA in the sample (Fig. 3). A third measurement then follows where another 20 μL of sample is injected but with the SAW device active, lysing the EVs. The subsequent CVC and voltage shift reflect the total, both EV-miRNA and free-floating, target miRNA concentration. Comparison between these two measurements leads to an accurate quantification of the target miRNA in EVs. Both of these CVCs take ∼30 min to complete, such that in ∼1 h, the integrated device can distinguish between the free-floating and target EV-miRNAs using only 40 μL of plasma. Furthermore, the IEM sensor is disposable^28^ and can be replaced after a single use to maintain the optimized repeatability of results.

### Performance of the platform with human plasma samples

**Fig. 4:**
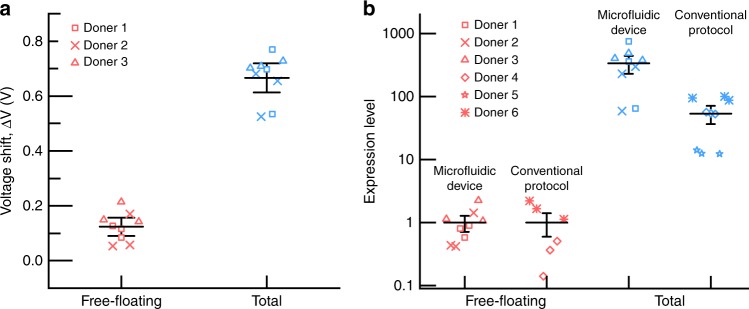
Characterization of free-floating and total miR-21 in healthy human plasma using the integrated microfluidic platform and benchmarked against conventional protocol using RT-qPCR. **a** The voltage shift Δ*V* for each analysis both before (free-floating) and after (total) SAW-induced EV lysis showing consistent results across the three donors. **b** Free-floating and total miR-21 expression level in healthy human plasma using both the integrated microfluidic platform and RT-qPCR protocol. For the expression level, the mean free-floating value measured by both the integrated device and RT-qPCR has been set to 1.0, and lysed exosome measurements are referenced to these values, respectively. Two different donor cohorts, each consisting of three healthy human plasma samples, were used for the microfluidic platform (donors 1–3) and RT-qPCR protocol (donors 4–6), respectively. For both panels, the mean reflects the average of all nine data points, and the error bars represent the uncertainty at 95% confidence

To assess the clinical efficacy of the device and method, a series of measurements were conducted on the plasma of three healthy human subjects. For each subject, three runs were conducted such that the total number of runs across the three subjects was nine. Each run consisted of analyzing both the free-floating and total miR-21. The plasma was tested ‘as is’ (see “Methods”) and no additional sample preparation was conducted. Voltage shifts were measured to be in the same range for all three different donors suggesting the conformity of miRNA concentration in healthy human plasma (Supplementary Fig. 3). The tests were conducted across three different fabricated integrated devices, illustrating the repeatability in both the fabrication and analysis (Supplementary Fig. 4). Figure 4a shows the compiled results for the voltage shift from all runs both before (free-floating) and after (total) lysing. As expected, the voltage shift increases after lysing due to the release of EV-miRNAs.

To ensure that the increase in signal was due solely to the miR-21 released by lysed EVs, a series of negative controls were conducted. The first was to investigate the response of a bare (nonfunctionalized) IEM sensor when exposed to plasma, and it did not produce a measurable voltage shift when exposed to either nonlysed or SAW-lysed plasma (Supplementary Fig. 5). A second test diluted the plasma in half using 1× PBS buffer, reducing the voltage shift from 0.65 V to 0.6 V as anticipated (Supplementary Fig. 6). Hybridization of the oligoprobe with nontarget miRNA was studied by spiking the plasma sample with 1 µM of nontarget miR-196a, and Supplementary Fig. 7 shows that a negligible voltage shift of 0.1 V was measured. Finally, a man-made probe with no known miR complements was used to functionalize the IEM sensor. For both unlysed and SAW-lysed plasma, a voltage shift of 0.08 V was measured (Supplementary Fig. 8), which is below the measurement limit of the IEM sensor (Fig. 2b).

To demonstrate that the voltage shift is indeed due to free-floating miR, we conducted the following control experiment. We first treated the plasma sample with RNAse to digest the free RNAs, and then the sample was injected into the integrated device. We did not measure any target miR-21, thus suggesting that the voltage shift measured for the unlysed plasma sample is due to free-floating target miR-21 (Supplementary Fig. 9). Since SAW lysing releases the contents of EVs, including negatively charged proteins, we also conducted a control experiment to ensure that the measured voltage shift is not affected by protein fouling. Protease was added to a SAW-lysed plasma sample to eliminate the proteins before introducing the sample to the IEM sensor; a similar voltage shift was measured as with a nonprotease-treated sample (Supplementary Fig. 10), thus further validating the specificity of the IEM sensing system against protein interference. As all these negative control tests (discussed in more detail in the Supplementary Notes) clearly demonstrated the high selectivity of the integrated device, we did not perform any control experiments with RNAse-treated SAW-lysed EV samples.

### Comparison to conventional analysis using RT-qPCR

In order to confirm the measured miR-21 concentration, the data were benchmarked against the conventional analysis method consisting of exosome isolation, chemical lysing, miRNA extraction, and quantitative RT-qPCR (see Methods) using three additional human plasma samples. All the data are normalized by the average free-floating miR-21 expression level. As shown in Fig. 4b, both our integrated platform and the conventional method produce similar results even when using two different donor cohorts. These results show that the EV-miR-21 are much more abundant than free-floating miR-21, consistent with the findings of others^36^. Notably, our platform uses far less sample (20 µL versus 1 mL) and requires less time (30 min versus 13 h) than RT-qPCR.

However, there are quantitative differences between the two approaches. The total miR-21 expression level measured using the integrated device is higher than the conventional RT-qPCR protocol. This could be attributed to the higher lysing efficiency of SAWs in our integrated device compared to the chemical (TRIzol^®^) lysis during the miRNA extraction step in the conventional method. To check this, we measured the miR-21 concentration using RT-qPCR for a sample that went through the complete standard protocol and a second sample that underwent an additional SAW lysing step prior to RT-qPCR. Supplementary Fig. 11 shows that the miR-21 expression level was 50% greater for the SAW-lysed plasma sample, which suggests the efficiency of SAW-based exosome lysing is 50% greater than TRIzol^®^-based lysis, accounting for the discrepancy in Fig. 4b. In order to validate the enhanced EV lysing is due to SAW, we performed the following control experiment. We chemically lysed a plasma sample using a commercial kit (Qiagen) and detected the target miR-21 using our concentration/sensing chip. A detectable voltage shift demonstrated successful detection of the miR-21 target. However, the voltage shift of the chemically-lysed plasma is almost 46% less than the SAW-lysed sample, confirming the superior lysing efficiency of the SAW device (Supplementary Fig. 12).

### Analysis of miR-21 biomarker in mouse models

**Fig. 5:**
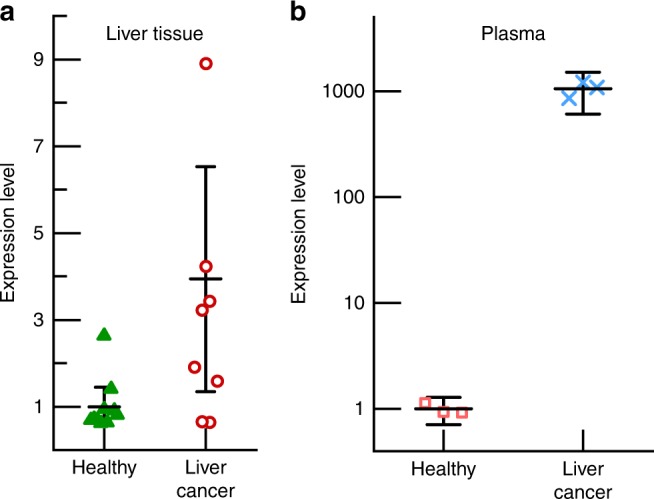
Measurement of miR-21 expression **a** in liver tissues using qPCR and **b** in plasma using the integrated device for two cohorts of 15-month-old mice, one healthy and one with liver cancer. In plasma, the expression level for the liver cancer group is nearly three orders of magnitude greater than that of the healthy (control) group. For the liver tissue data, the mean reflects the average of ten (healthy) and nine (liver cancer) replicates respectively, and for the plasma, both data sets show the average of three replicates. The error bars represent the uncertainty at 95% confidence in all cases

Having established the ability of the integrated platform to detect miR-21 in human plasma, we next evaluated its efficacy for detecting the presence of tumors that overexpress miR-21. We chose to use a genetically-engineered liver cancer mouse model, since the presence of tumors could be confirmed by pathological examination and miR-21 is considered to be a potential biomarker^36,37^. We used a mouse model where liver cancer was generated by conditional deletion of the tumor suppressor gene *Pten* (phosphatase and tensin homolog deleted on chromosome 10) in hepatic cells^38^. All mice that carry deletion of *Pten* develop spontaneous liver cancer by 12 months of age and these tumors express high miR-21 as shown in Fig. 5a.

The plasma from two cohorts of two mice (15 months old) was analyzed. One group was a control containing healthy mice, and the other group contained mice with *Pten* deletion that developed liver cancer. The plasma samples were analyzed in a blind-fashion. Figure 5b shows the total miR-21 concentration in plasma for the two cohorts of mice. Notably, the expression level of total miR-21 in the mice from the liver cancer cohort is elevated around three orders of magnitude when compared to the level of miR-21 in the plasma of mice from the healthy cohort. This is consistent with the observation that miR-21 is upregulated in the tumors developed in these mice (Fig. 5a) as the plasma samples in Fig. 5b came from the same mice used for Fig. 5a. It is notable, however, that the upregulation of miR-21 for liver tissue (Fig. 5a) is only ~3 times, while it is close to 1000 for EVs in plasma measured by our integrated microfluidic device (Fig. 5b), which others have also observed for serum^36^. This discrepancy could arise from the absence of EVs in tissues to protect the target miR from degradation. It could also be due to analyte loss during the multiple steps in the tissue test. Finally, the superior sensitivity of our platform compared to the conventional techniques, shown in Fig. 4, could also be a factor. This finding demonstrates the efficacy of this integrated platform for diagnostic and prognostic applications.

### Analysis of miR-21 biomarker in clinical human samples

**Fig. 6:**
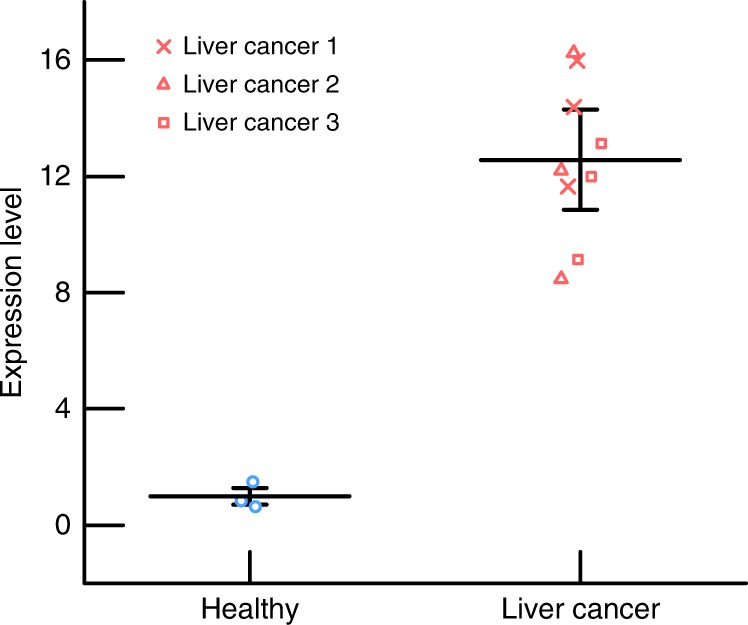
Measurement of miR-21 expression in human serum samples using the integrated device for two cohorts, one healthy and one with liver cancer. In serum, the expression level for the liver cancer group is nearly 13 times greater than that of the healthy (control) group. For healthy and liver cancer groups, data sets show the average of three and nine data points, respectively. The error bars represent the uncertainty at 95% confidence in all cases

To test the ability of the integrated systems to analyze clinical samples, serum from healthy patients and patients confirmed to have liver cancer were both tested. After SAW lysing of serum samples, the concentration of miR-21 was measured for both cohorts. Figure 6 shows that there is a nearly 13-fold overexpression of miR-21 in serum samples from the patients with liver cancer when compared with healthy human serum. Further, there is no overlap with the healthy group, confirming the ability of our integrated device to detect cancer in clinical samples. As the platform successfully identified the overexpression of miR-21 from liver cancer patients, we limited our validation study to three clinical samples; future studies will focus on a more comprehensive clinical evaluation.

## Discussion

In this work, we present a single integrated device that streamlines the analysis of EV-miRNAs from plasma, requiring less than one-tenth of the time and sample than conventional RT-qPCR methods. The device achieves detection levels down to 1 pM using only 20 µL of sample with an assay time of 30 min. This sensitivity is sufficient for accurate PCR-free quantification of miR-21 from untreated plasma or serum samples, as verified with our clinical sample studies. Across multiple samples, we measured two orders of magnitude overexpression of EV miR-21 over their free-floating counterpart. To the best of our knowledge, this represents the first accurate quantification of free-floating and EV-miRNAs. The potential advantages of this integrated device are clear. Unlike conventional RT-qPCR approaches, this new integrated system does not require pretreatment or analyte transfer. Due to these analyte loss prevention features, the integrated device produces fast (30 min), reliable, and repeatable absolute miRNA quantification from a small volume (20 µL) of untreated sample with 10% uncertainty.

Our IEM sensor works on the principle of charge inversion phenomenon and detects the presence of negative charge on target molecules. Hence, the integrated device can be easily extended to detect other cancer biomarkers including protein biomarkers. We have already successfully tested this platform against a number of different targets such as short ssDNA molecules, miRNA molecules associated with pancreatic cancer (miR-550)^25^ and oral cancer (miR-146a) as well as RNA from dengue virus, Brucella bacteria, and *Escherichia coli* (E. coli).^28^ This clearly shows the potential of the platform for analyzing different cancer biomarkers. As protein biomarkers are weakly charged compared to nucleic acids, an ELISA scheme can be used where a secondary antibody reporter attached to a negatively charged molecule can be designed to introduce the necessary negative charge to the antibody–antigen complex so that a change in the CVC can be registered when the target protein biomarkers are captured. In addition, the integrated platform can be easily multiplexed by fabricating multiple sensors close to each other so that a panel of cancer biomarkers can be simultaneously detected from clinical samples. The successful development of the multiplexed integrated device would offer the first liquid biopsy diagnostic device that is simple, rapid, and user-friendly capable of detecting both miRNAs and protein target biomarkers from clinical samples.

## Methods

### Solvents, miRNA, and oligonucleotide probes

Two buffers, PBS and 10× TAE buffer consisting of 400 mM Tris-acetate and 10 mM ethylenediaminetetraacetic acid (EDTA), were purchased (Fischer Scientific) and used as is. Ethanol was purchased (pure, Sigma-Aldrich) and used as is.

The target miRNA hsa-mir-21-5p (miR-21) has a base sequence given by TAGCTTATCAGACTGATGTTGA^39^. To establish the calibration curve for the IEM sensor, miR-21 was purchased (Integrated DNA Technologies) and used as is. For all studies IEM sensors were functionalized with the complimentary sequence of miR-21, an amine-coupled ssDNA oligonucleotide probe with a sequence of TCAACATCAGTCTGATAAGCTA (Integrated DNA Technologies). For further studies and performing negative control tests, a ssDNA sequence, TAGGTAGTTTCATGTTGTTGGG of hsa-miR-196a-5p, was purchased (Integrated DNA Technologies) and used as a sample. In another study, a man-made amine-coupled ssDNA sequence, GATCGCAGCCAAATGACGTGAC was purchased (Integrated DNA Technologies) and used as an oligo probe. The *Caenorhabditis* *elegans* miR-39 miRNA mimic was purchased (Qiagen) and used as a spiked-in control when conducting the miRNA extraction during the conventional analysis protocol.

### Healthy human and mouse model plasma samples

#### Healthy human samples

Human donor samples were purchased (Zen-Bio Inc.) and consisted of 10 mL of fresh human plasma collected in tubes with EDTA coagulant. All samples were obtained following FDA-mandated testing for pathogens.

#### Mouse model samples

Mice carrying the targeted deletion of *Pten* in hepatic cells were generated as previously reported^38^. The tumor mice were 15-month-old male *Pten*^*loxP/loxP*^; *Alb-Cre*^+^ mice on C57/Bl6 background. All mice were housed in a temperature-, humidity-, and light-controlled room (12 h light/dark cycle), and were allowed free access to food and water. Plasma samples were collected from the 15-month-old mice via cardiac puncture as an end procedure. All experiments were conducted according to the Institutional Animal Care and Use Committee (IACUC) of the University of Southern California research guidelines and approved by the University of Southern California IACUC review committee.

#### RNA extraction and RT-qPCR from mouse tumors

Mouse livers were perfused in PBS and fresh frozen in liquid nitrogen until use. Frozen liver tissues were lysed in Trizol for total RNA extraction. RT-qPCR was performed using an Invitrogen first strand cDNA synthesis kit followed by qPCR analysis as previously reported^40^.

### Human liver cancer patient serum acquisition

De-identified human liver cancer samples were obtained under an approved IRB (for B.L.S.) from the University of Southern California tissue bank.

### Fabrication of the surface acoustic wave lysing chip

A 128° YX lithium niobite (LiNbO_3_) piezoelectric substrate was purchased (Precision Micro-Optic PWLN-431232), and 20 pairs of 200-nm-thick interdigitated aluminum electrodes were deposited using standard microfabrication techniques^41^ (using 20 nm titanium as an adhesive layer) to form what is known as a width-controlled, single-phase, unidirectional interdigitated transducer (IDT)^42^. The electrode width and spacing were designed to achieve a resonant frequency of 28 MHz, where the generated SAW propagates orthogonal to the IDT (Fig. 1b). A sinusoidal wave form was generated by a function generator (Agilent 33250A) and amplified by a radio frequency amplifier (E&I 325LA) to actuate the SAW device. For all results shown here, the SAW was operated at 28 MHz and 1 W.

A microfluidic channel was fabricated out of polycarbonate thermosoftening plastic in a manner similar to our previous work^27^. Two layers of polycarbonate were used to fabricate a 15 × 2 × 0.3 mm^3^ (*l/w/t*) microfluidic channel that was attached to the LiNbO_3_ substrate using double-sided Kapton^®^ tape. The microchannel was oriented parallel to the IDTs that generate the SAW such that the generated SAW was perpendicular to the microchannel (Fig. 1b).

### Fabrication of concentration/sensing chip

For the concentration/sensing chip, a three-layered polycarbonate-based microfluidic channel with dimension of 60 × 2 × 0.3 mm^3^ (*l/w/t*) was fabricated, and orifices for the inlet, outlet, sensing and concentration units were cut into the channel. The preconcentration unit is designed to collect all the negatively charged molecules, including the target miRNA, directly beneath the IEM sensor where the hybridization occurs. Two CEMs (Mega a.s., Czech Republic) were implanted at the bottom of the device to bridge between the main microfluidic channel and two electrode reservoirs through designed orifices (Fig. 1c). Two electrode reservoirs were filled with 10 × TAE buffer. Two platinum electrodes were inserted in the reservoirs and 200 V of direct current (DC) voltage was applied using a DC power supply (Keithley 2400 SourceMeter). Application of the voltage generates a depletion zone at the interface of the IEM and thus concentrates negatively charged species upstream of the depletion zone (Fig. 1c)^26^.

The IEM sensor is an anion-exchange nanoporous membrane, made of polystyrene–divinylbenzene fine particles with strong basic quaternary ammonium groups supported by polyethylene as a binder and polyamide/polyester textile fiber (Mega a.s., Czech Republic), which is framed by a resin mold^28^. It is placed in an orifice at the top of the primary microfluidic channel just upstream of the concentration IEM microchannel (Fig. 1c, d). The sensor is functionalized with the oligoprobe using our standard procedures^28^.

To obtain the current–voltage characteristics, a Gamry 500 potentiometer (Gamry Instrument) was used where an electric current is applied between the IEM sensor reservoir and the upstream reservoir via two platinum electrodes (Fig. 1d), and the voltage drop across the IEM sensor is measured between the sensor reservoir and a downstream reservoir by two reference electrodes (Ag–AgCl). The microfluidic channel and two electrode reservoirs always contain 0.1× PBS at the time of CVC measurements.

### Conventional protocol for miRNA lysis/extraction from plasma

miRNAs were isolated from plasma samples using a miRNeasy Serum/Plasma Kit (Qiagen) following the user manual. Five volumes (relative to the sample volume) of QIAzol Lysis Reagent (Qiagen) was added to the sample and mixed by vortexing. After incubation at room temperature for 5 min, 3.5 μL (1.6 × 10^8^ copies μL^−1^) of cel-miR-39-3p in RNase-free water was added to the lysing buffer as a normalization control. Chloroform at an equal volume as the plasma sample was added, and the tube was vortexed for 15 s. After placing at room temperature for 3 min, the mixture was centrifuged for 15 min. The upper aqueous phase was collected and mixed with 1.5 volumes of 100% ethanol. The resulting sample was pipetted into a RNeasy MinElute spin column (Qiagen) and centrifuged at 10,000 × *g* for 15 s. (If the mixture was more than 750 μL, then a 750 μL aliquot of the mixture was transferred and centrifuged first, and then the same was done for the remainder). The RNeasy MinElute spin column was washed with 700 μL Buffer RWT and 500 μL Buffer RPE (miRNeasy Serum/Plasma Kit, Qiagen) at 10,000 × *g* for 15 s, sequentially. Subsequently, 500 μL of 80% ethanol was pipetted onto the spin column, and the column was centrifuged at 10,000 × *g* for 2 min. After putting the spin column into a new collection tube, the column was centrifuged at 17,000 × *g* for 5 min to dry the membrane. Finally, miRNAs were eluted with 14 μL RNase-free water into a collection tube by centrifugation at 17,000 × *g* for 1 min.

### Quantification of miRNA concentration using RT-qPCR

Reverse transcription was carried out using a miScript II RT Kit (Qiagen). A 20 μL reverse transcription reaction was prepared with 2.2 μL of eluted miRNA, 4 μL 5× miScript HiSpec Buffer (Qiagen), 2 μL 10× miScript Nucleics Mix (Qiagen), 9.8 μL RNase-free water, and 2 μL miScript Reverse Transcriptase Mix (Qiagen). The reaction was incubated at 16 °C for 60 min followed by 95 °C for 5 min. The reverse transcription reaction was then diluted with 200 μL RNase-free water. Triplicates of qPCR reactions were carried out using miScript SYBR Green PCR Kit (Qiagen) and run on a StepOnePlus™ Real-Time PCR System (Applied Biosystems). The reaction contained 2 μL diluted cDNA, 12.5 μL 2× QuantiTect^®^ SYBR Green PCR Master Mix (Qiagen), 2.5 μL 10× miScript Universal Primer (Qiagen), 10× miScript Primer Assay (Qiagen) for the target miRNA, and 5.5 μL RNase-free water in a final volume of 25 μL. The reaction mixtures were incubated for 15 min at 95 °C, followed by 45 cycles of 94 °C for 15 s, 55 °C for 30 s, and 70 °C for 30 s. Standard curves were generated from a series of dilutions for the target miRNA (synthetic miScript miRNA Mimics from Qiagen) for each plate (Supplementary Fig. 13). Quantitation cycle (*Cq*) values were acquired and analyzed using StepOne™ Software v2.3 in accordance with the MIQE guidelines^43^.

### EV Isolation for comparing free-floating and EV-miRNA

EVs were isolated from 1 mL plasma samples using an ExoQuick^®^ Plasma Prep and EV precipitation kit (System Biosciences Inc.) according to the manufacturer’s instruction. Twelve microlitres thrombin at 500 U mL^−1^ was added to the plasma sample and mixed by flicking several times. After incubation at room temperature for 5 min and centrifugation at 10,000 × *g* for 5 min, the supernatant, which contained free-floating miRNA and EVs, was taken and treated with the 215 μL ExoQuick™ Exosome Precipitation Solution (System Biosciences Inc.) and incubated for 1 h at 4 °C. The mixture was then centrifuged at 1500 × *g* for 30 min, and the supernatant containing free-floating miRNA was collected and diluted to 1 mL with 1× PBS. The pelleted EVs were resuspended in 1 mL 1× PBS. Two-hundred microlitres of each sample (diluted supernatant, resuspended EVs, and original plasma sample) was used in miRNA extraction and RT-qPCR analysis following the aforementioned methods.

### Expression level calculation

The expression level of certain miRNA is often presented as the ratio between the target miRNA and some reference. For the RT-qPCR analysis, the expression level is calculated as

$${\mathrm{Expression}}\,{\mathrm{level}} = {\mathrm{2}}^{\left( {{\textstyle{{Cq_{{\mathrm{target,sample}}} - Cq_{{\mathrm{target,reference}}}} \over {b_{{\mathrm{target}}}}}}} \right)}$$

where *Cq* is the quantitation cycle and

$$b_{{\mathrm{target}}} = - {\mathrm{slope}}\,{\mathrm{of}}\,{\mathrm{the}}\,{\mathrm{standard}}\,{\mathrm{curve}}/\log _210$$

If extraction was included, the expression level was further normalized with the spiked-in normalization control (in this case miR-39) using

$${\mathrm{Expression}}\,{\mathrm{level}} = {\mathrm{2}}^{\left( {\frac{{\,Cq_{{\mathrm{target,sample}}} - Cq_{{\mathrm{target,reference}}}}}{{b_{{\mathrm{target}}}}} - \frac{{Cq_{{\mathrm{spike}} - {\mathrm{in,sample}}} - Cq_{{\mathrm{spike}} - {\mathrm{in,reference}}}}}{{b_{{\mathrm{spike}} - {\mathrm{in}}}}}} \right)}$$

For the microfluidic platform, the voltage shifts Δ*V* from the CVCs can also be transformed into a relative expression level using

$${\mathrm{Expression}}\,{\mathrm{level}} = {\mathrm{exp}}\left( {\frac{{\Delta V_{{\mathrm{target}}} - \Delta V_{{\mathrm{reference}}}}}{b}} \right),$$

where *b* is a fitting parameter extracted from the calibration curve (Fig. 2b) and Δ*V*_reference_ is the relative voltage shift for a desired reference (e.g., the voltage shift for the case of free-floating miRNA).

### Statistics

For this work, we employed standard uncertainty analysis^40^ where the precision uncertainty was calculated using

$$u = t_{n - 1,95{\mathrm{\% }}}\sigma /\sqrt n$$

where *n* is the number of experimental replicates, *σ* is the standard deviation of the data set, and *t*_*n* − 1,95%_ is the two-tailed Student’s *t* value for *n* − 1 degrees of freedom at 95% confidence. This was applied to all data sets and calculated and plotted using the software OriginPro^®^.

Figure 2b and Supplementary Figs. 1a–c, 3, 7, 8, 9, 10, and 13 show plots where the data points reflect mean values and the error bars reflect the uncertainty intervals at 95% confidence. For Supplementary Fig. 1a, five replicates were conducted (*n* = 5) such that the degrees of freedom were *n* − 1 = 4 and the *t* value was *t*_4,95%_ = 2.776. For all the other figures, three replicates were conducted (*n* = 3) such that the degrees of freedom were *n* − 1 = 2 and the *t* value was *t*_2,95%_ = 4.303.

Figures 4 and 5b and Supplementary Figs. 2, 4, and 11 show scatter of the raw data along with mean values with error bars reflecting the uncertainty intervals at 95% confidence. In both Fig. 4 and Supplementary Fig. 4, three replicates were conducted for each plasma sample from each of the three healthy donors such that there were *n* = 9 data points for each condition. The plot of the mean value reflects the average of the *n* = 9 data points, and the error bars correspond to *n* − 1 = 8 degrees of freedom and a *t* value of *t*_8,95%_ = 2.306. For Fig. 5 and Supplementary Figs. 2 and 11, three replicate experiments were conducted for each condition (*n* = 3) such that the degrees of freedom were *n* − 1 = 2 and the *t* value was *t*_2,95%_ = 4.303.

Figure 5a shows scatter of the raw data along with mean values with error bars reflecting the uncertainty intervals at 95% confidence for RT-qPCR analysis of liver tissue from the healthy and liver cancer mouse models. Quantitation of the relative expression level was done using the delta-delta-Ct (ddCt), where five healthy samples were each analyzed twice (*n* =10) and five liver cancer samples were each analyzed twice, save for one that was only analyzed once (*n* = 9). (Note that these *n* values are after removing outliers.) The degrees of freedom were *n* − 1 = 9 and *n* − 1 = 8, respectively with *t* values of *t*_2,95%_ = 2.228 and *t*_2,95%_ = 2.262. For comparison, the data were normalized to the mean of the healthy results.

### Reporting summary

Further information on research design is available in the Nature Research Reporting Summary linked to this article.

## Supplementary information

Supplementary Information

Description of Supplementary Data

Reporting Summary

Supplementary Data 1

## Data Availability

The authors declare that the main data supporting the findings of this study are available within the article, and data sets for the main figures are in Supplementary Data 1. Additional data, such as for supporting figures, are available from the corresponding author D.B.G. upon request.
