# Supplementary material for: Extracellular vesicle microRNA quantification from plasma using an integrated microfluidic device
Source: Commun Biol. 2019 May 20;2:189. doi: 10.1038/s42003-019-0435-1 (PMC6527557; doi:10.1038/s42003-019-0435-1)
Supplement: Supplementary file 1 — Supplementary Information [file 42003_2019_435_MOESM1_ESM.pdf]

Supplementary Figures

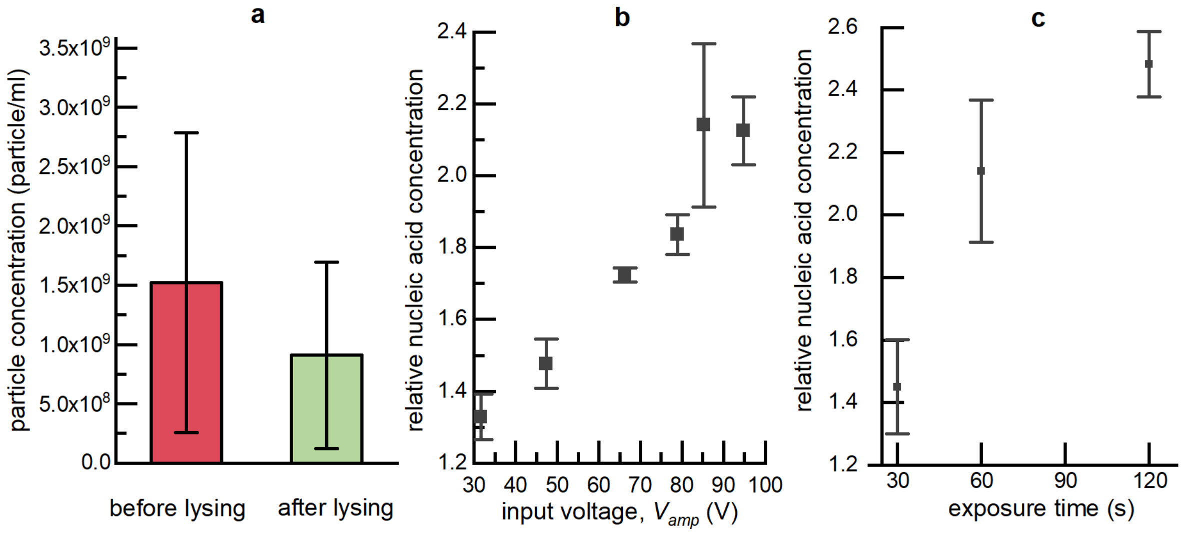

Supplementary Figure 1 Characterization and optimization of SAW device operation. (a) NanoSight™ measurements of 30 nm to 150 nm nanoparticles from an isolated EV sample both before and after SAW lysing at 1000 µl hr^{-1} and 80 V_{amp}. For each condition, 5 experiments were conducted and the bar plot reflects the mean with error bars at 95% confidence. The relative increase in the nucleic acid concentration as measured by a NanoDrop™ instrument as a function of (b) the input voltage to the SAW device and (c) the exposure time of the sample to SAWs as determined by the flow rate. Lysing efficiency can be inferred by measuring the relative increase in the nucleic acid concentration in solution. At sufficiently high voltages and exposure times, the increase plateaus, reflecting near 100% lysing. Each data point reflects 3 experiments and uncertainty at 95% confidence.

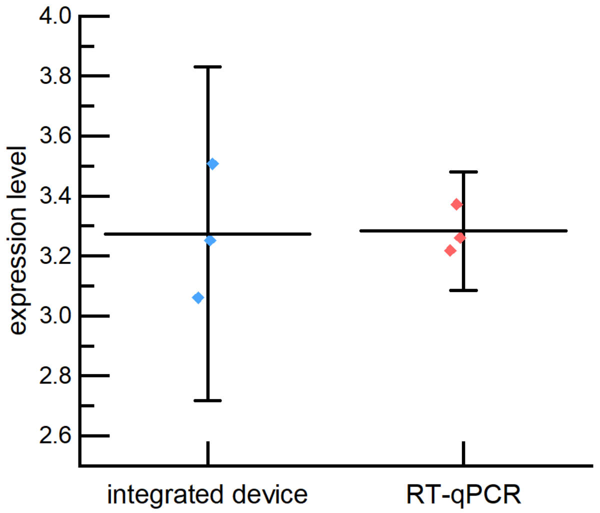

Supplementary Figure 2 miR-21 expression level for a 50 pM solution of synthetic miR-21 in 1×PBS as determined by the integrated device and RT-qPCR. The error bars indicate three replicates at 95% confidence.

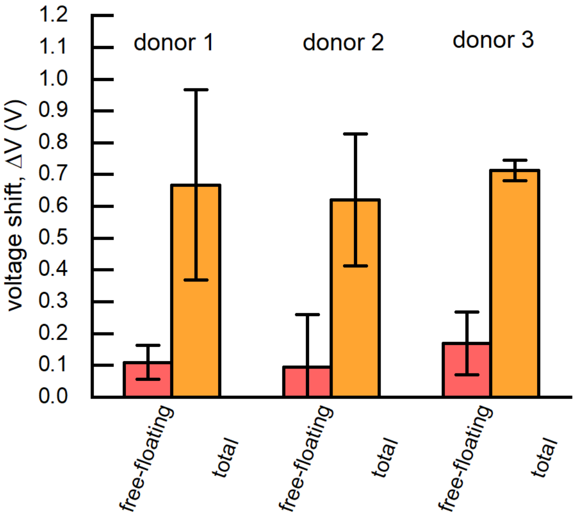

Supplementary Figure 3 Analysis of plasma samples from three healthy human donors. The voltage shifts of both non-lysed (free-floating) and SAW-lysed (total) samples are shown. For each subject, 3 runs were conducted, and mean and uncertainty at 95% confidence are shown.

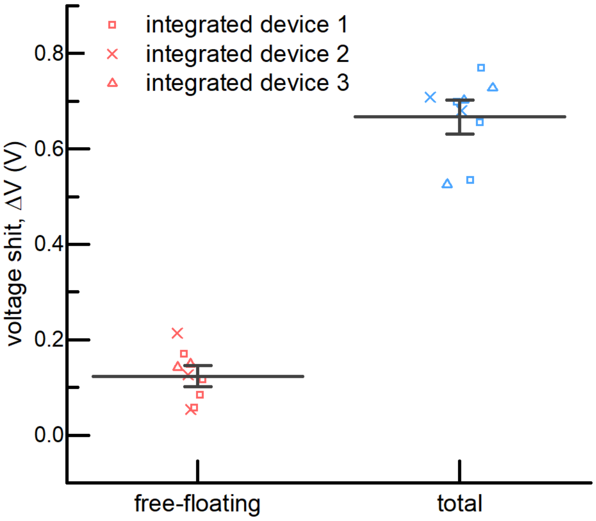

Supplementary Figure 4 Comparison of three different integrated devices. The measured voltage shifts ΔV before (free-floating) and after (total) SAW lysing using three different fabricated integrated devices. For each device, 3 runs were conducted both prior to and after lysing. The average of all measurements and across all devices is also shown as well as uncertainty intervals at 95% confidence.

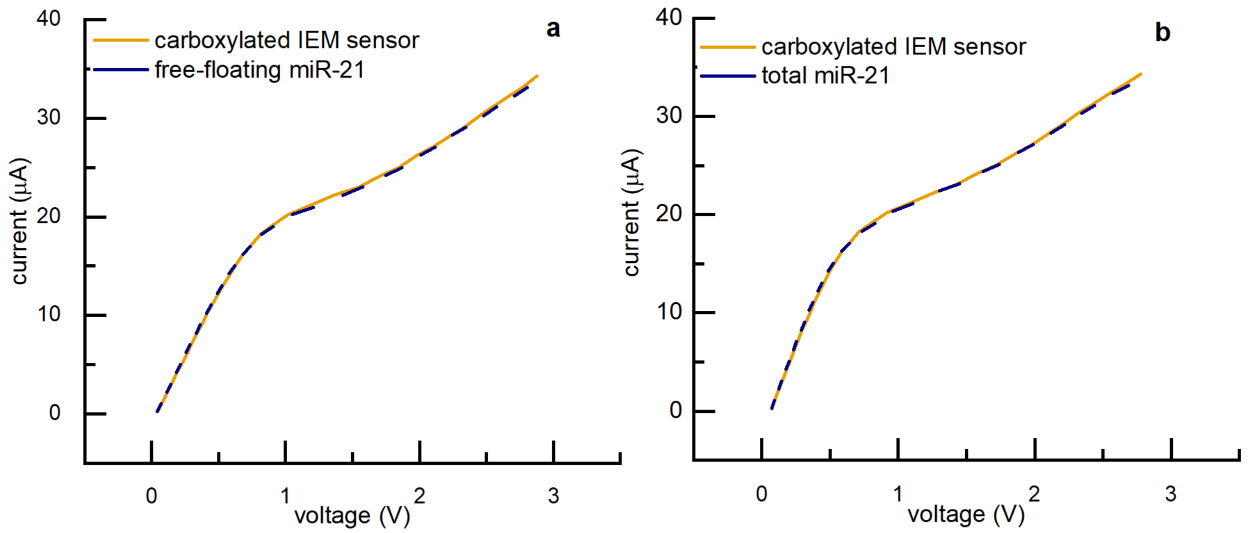

Supplementary Figure 5 Performance of a carboxylated IEM sensor with no probe attached. The measured CVCs for the carboxylated IEM sensor in 1×PBS and (a) 20 µL of non-lysed human plasma and (b) 20 µL of SAW-lysed human plasma. The non-lysed plasma sample contains free-floating miR-21 and the SAW-lysed sample contains the total miR-21 content.

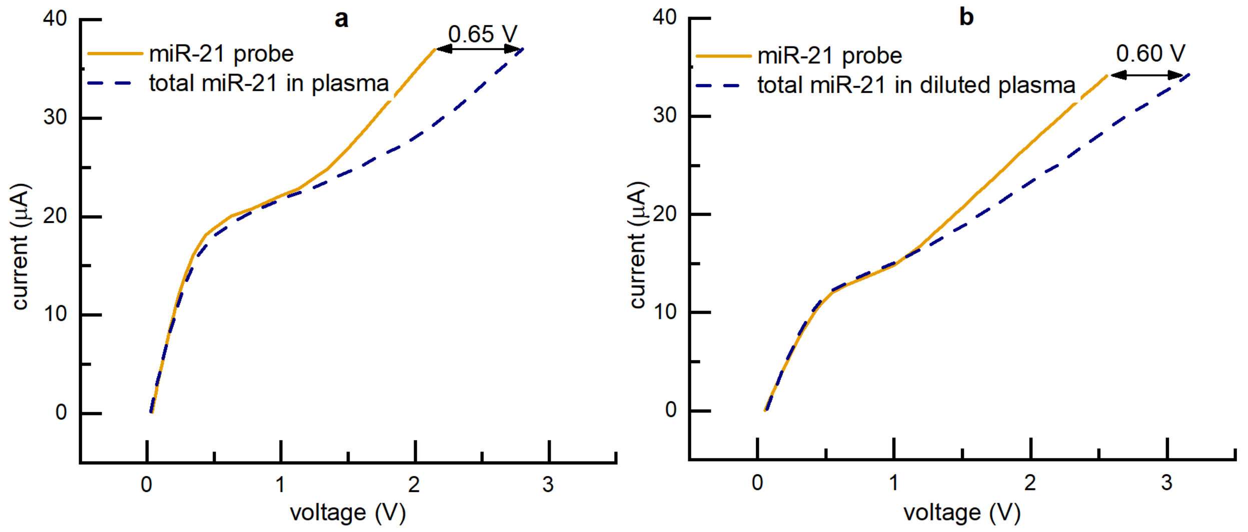

Supplementary Figure 6 Effect of plasma dilution on the voltage shift. Measured CVCs for the (a) total miR-21 in healthy human plasma and (b) total miR-21 in healthy human plasma diluted by 50% using 1×PBS.

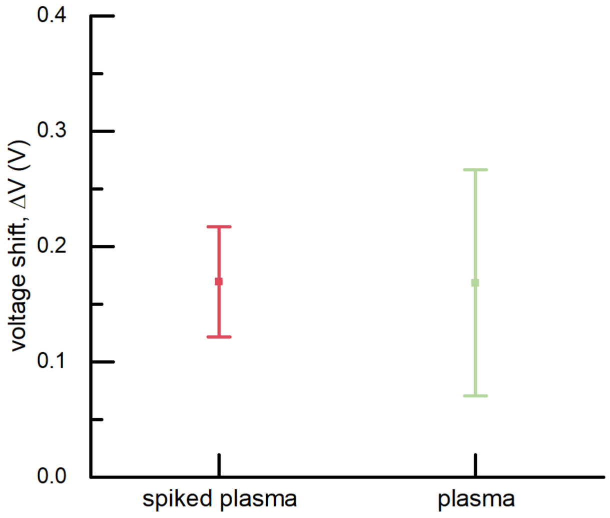

Supplementary Figure 7 Comparison of a plasma control and plasma spiked with 1 µM of miR-196a to test for non-specific binding. Almost no change in sensing signal was measured when compared with measured free-floating miR-21 in plasma.

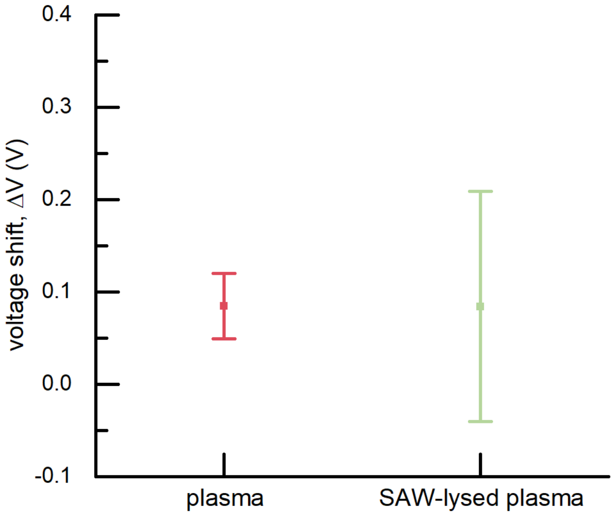

Supplementary Figure 8 Integrated device results for free-floating and total miR-21 in healthy human plasma, when the membrane sensor is functionalized using a man-made oligo probe with no known miR complements. Voltage shifts are negligible.

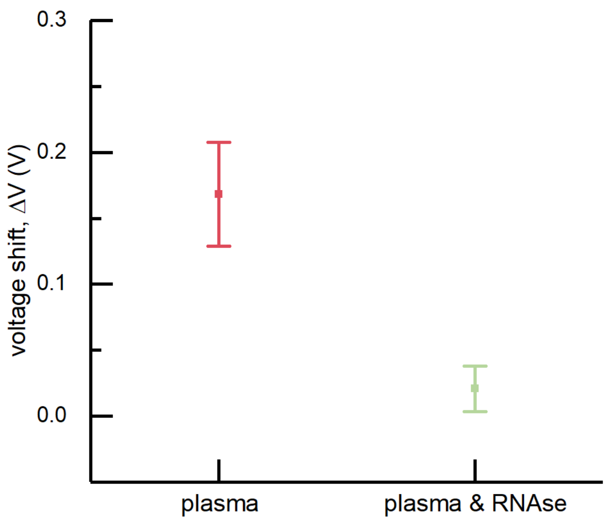

Supplementary Figure 9 Voltage shift for plasma and plasma with RNAse. After digesting the free-floating miRNAs using RNase, almost no voltage shift was measured by the integrated device, confirming that free-floating target miRNA are in fact being detected.

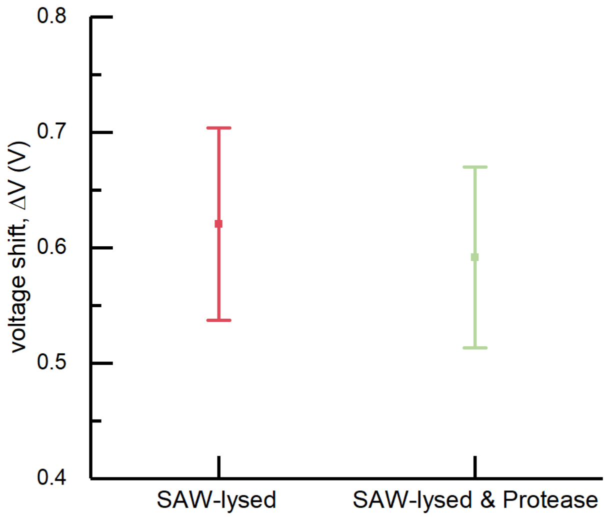

Supplementary Figure 10 Voltage shift when protease is added to a SAW-lysed plasma sample. By eliminating the released proteins from the SAW-lysed plasma sample, specificity of the functionalized IEM sensor towards miR-21 was investigated. Voltage shifts are almost equal, indicating no adverse interference from proteins released during SAW lysis.

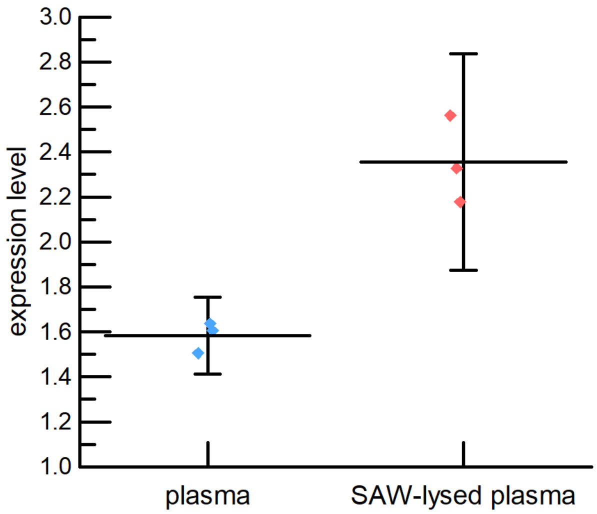

Supplementary Figure 11 Comparison of SAW lysing to TRIzol®-based lysing using RT-qPCR. The measured miR-21 expression levels with and without SAW lysis are shown with a measured concentration 50% higher when using SAW lysis. For each case, 3 runs were conducted, and mean and uncertainty at 95% confidence are shown.

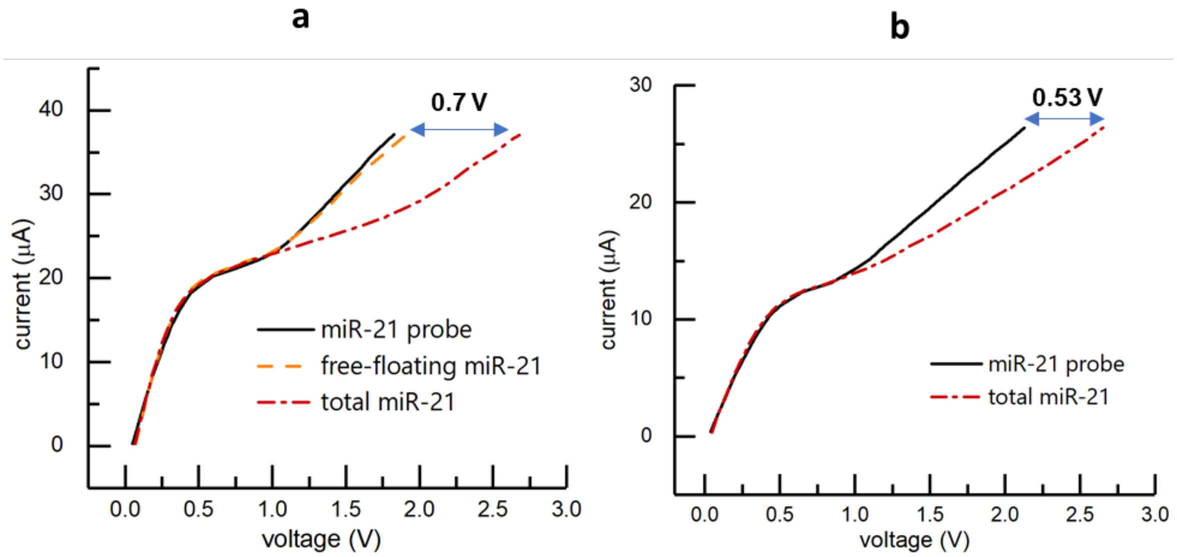

Supplementary Figure 12 Comparison of SAW lysing to TRIzol®-based lysing using the sensing device. Measured CVCs for the (a) free-floating and total miR-21 in SAW-lysed healthy human plasma and (b) total miR-21 in chemically-lysed healthy human plasma. The miR-21 expression level was 46% lower using the commercially-available chemical lysis kit compared to the SAW device.

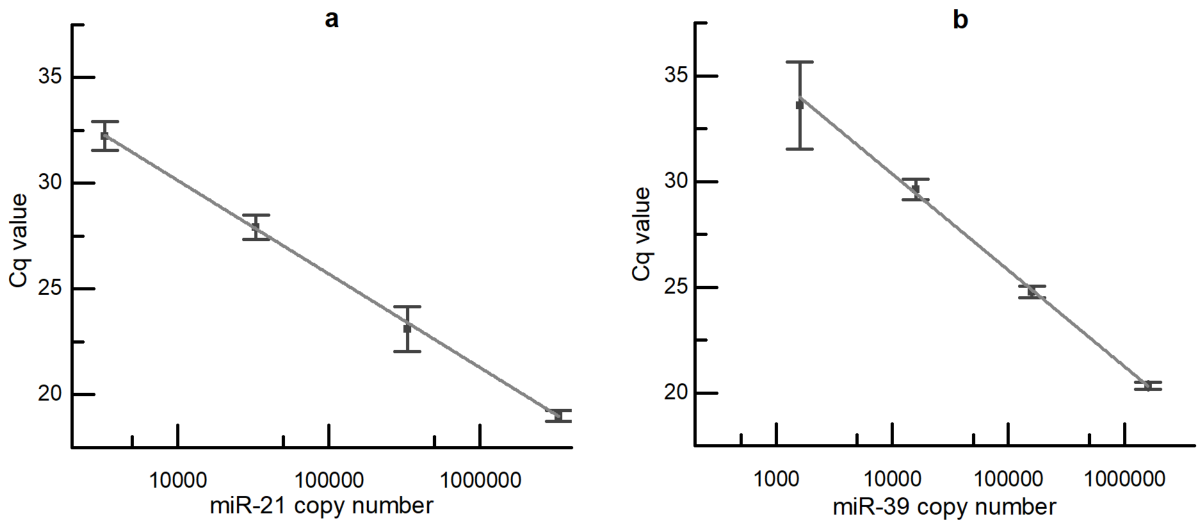

Supplementary Figure 13 Standard RT-qPCR curves for (a) miR-21 and (b) miR-39.

Supplementary Notes

Characterization of SAW-based EV Lysing

The efficiency and performance of SAW lysing of extracellular vesicles (EVs) was carried out on isolated exosome samples. Exosome samples were isolated from AsPC-1 cells grown in culture using Exoquick (System Biosciences). For these studies, a 20 µL isolated exosome sample was injected into the microfluidic channel attached to the SAW device and exposed to SAW under various flow rates and operating voltages.

Two measurements were used to estimate the lysing performance by comparing the results prior to and after exposure to SAWs. A NanoSight™ nanoparticle tracking instrument (Malvern Panalytical) was used to count the exosomes in the samples, and five measurements were performed before and after SAW lysing. We specifically focused on nanoparticles with measured diameters between 30 nm to 150 nm since exosomes, which are small extracellular vesicles (EVs), are known to be in this size range and less than 200 nm[1,2,3]. Supplementary Figure 1a shows a 40% decrease in the number of small EVs in that size range after SAW exposure for 30 seconds (flowing through the lysing chip at 1000 µL hr^{-1}) at an actuation voltage of 80 V_{amp}. While using the NanoSight™ instrument demonstrates the efficacy of small EV lysing, it is not an accurate estimation of the number of lysed small EVs since lysing does not require the complete destruction

of a small EV; it is possible for lysing to occur by generating only a small fracture on the lipid membrane surface[4].

As a second characterization, the nucleic acid concentration in the sample was measured before and after SAW lysing using a NanoDrop™ 2000 UV-Vis spectrophotometer (Thermo Scientific); the relative increase of nucleic acid concentration in the solution is indicative of small EVs being lysed to release their miRNA and mRNA regardless of whether the small EVs are completely destroyed. Supplementary Figure 1b shows the relative increase in the solution concentration of nucleic acid as the SAW applied input voltage was increased from 30 to 95 V_{amp} (corresponding to an increase in the SAW amplitude) at a fixed flow rate of 500 µL hr^{-1}. The data reflect the mean of three measurements before and after SAW lysing. Not surprisingly, the nucleic acid concentration increased essentially linearly with SAW amplitude until saturating at approximately 85 V_{amp}, which is indicative of nearly complete lysing of the small EVs in the solution. Similarly, Supplementary Fig. 1c shows the relative increase in nucleic acid concentration as the exposure time to SAWs increases by increasing the flow rate from 250 µL hr^{-1} to 1000 µL hr^{-1} at a constant applied input voltage of 80 V_{amp}; again, greater exposure increased SAW lysing. Design of the operating conditions for the final integrated device was based on setting the flow rate and flow duration of the sample to ensure near complete lysing, where the limitations were practical issues, such as cavitation induced by the SAW in the microchannel.

miRNA Quantification Comparison between the Integrated Platform and RT-qPCR

We also conducted a study to compare the miRNA quantification characteristics between our integrated platform and conventional RT-qPCR protocol. Using synthetic miR-21 (miScript miRNA Mimics, Qiagen), two different concentrations (10 pM and 50 pM) of synthetic miR-21 solution in 1×PBS were tested both with the integrated device and conventional RT-qPCR. For RT-qPCR, the samples were added into the reverse transcription reaction directly without extraction. Supplementary Figure 2 shows the miR-21 expression level for the 50 pM solution relative to the 10 pM solution using both approaches, with the expression level defined in the Methods section of the manuscript. Notably, both techniques produced nearly identical mean values for the expression level, with the integrated device having slightly greater scatter in the

data. It should be noted that the smaller error bars obtained using RT-qPCR is due to the use of a purified synthetic miRNA sample without employing any sample purification.

Integrated Device Performance on Three Healthy Human Plasma Samples

Supplementary Figure 3 shows the voltage shift for healthy human plasma samples, gathered from 3 different donors, with and without SAW lysing. The voltage shift after SAW lysing is due to hybridization of both free-floating and released EV miR-21; it is almost 6 times greater than the voltage shift before EV lysing. Despite the small difference between the voltage shifts for different plasma samples, SAW lysing increased the voltage shift by at least a factor of 5 for all samples indicating the abundance of miR-21 inside plasma EVs. Additionally, voltage shifts recorded by the integrated device were measured to be almost in the same range for all 3 different samples suggesting the conformity of miRNA concentration in healthy human plasma.

Consistent Performance Across Multiple Devices

To verify that measurement performance was repeatable across different integrated devices, three different integrated devices were fabricated and used to measure the free-floating and total miR-21 content in healthy human plasma samples. As shown in Supplementary Figure 4, each device produced roughly the same results, with no discernible bias.

Negative Controls in Analysis of Human Plasma Samples

In order to verify the reliability of the miRNA quantification results on human plasma samples, a series of negative controls were designed and implemented as discussed below. In all cases, the negative controls showed that an IEM sensor functionalized with a miR-21 probe is specific to miR-21 content in the plasma and not affected by any other plasma bioparticles or non-specific binding.

(i) Performance of non-functionalized IEM sensor

In order to test whether human plasma artificially affected the behavior of an IEM sensor, we tested an IEM sensor that was only carboxylated but not functionalized with any miRNA probe(s). In principle, it should not have any response to human plasma since no miRNA will bind to the IEM. Both 20 μL of plasma and SAW-lysed plasma from a single patient were introduced to the sensor in the integrated device. Supplementary Figure 5a shows the measured CVC for the carboxylated IEM sensor alone in 1×PBS and then after being exposed to pure (non-lysed) human plasma containing free-floating miRNA. Similarly, Supplementary Fig. 5a shows the measured CVC for the carboxylated IEM alone and then after being exposed to SAW-lysed human plasma containing the total (free-floating plus EV) miRNA in the plasma. In both cases, the difference between the 1×PBS buffer and the plasma sample CVCs is negligible (approximately 0.001 V), clearly indicating that no bioparticles in the plasma attach to the IEM in the absence of a miRNA probe.

(ii) Effect of plasma dilution on IEM sensor response

To examine the IEM sensor sensitivity to miR content and ensure the voltage shift is due solely to the miR-21 in the plasma, the IEM sensor was carboxylated and functionalized with the miR-21 probe as described in the Methods section. 10 μL of SAW-lysed plasma diluted with 10 μL of 1×PBS was injected into the integrated device, and the voltage shift was measured before and after sample injection. As shown in Supplementary Figure 6, the voltage shift decreases with dilution, which can be attributed to the reduced concentration of miR-21 in the diluted sample. Using the miR-21 calibration curve, the concentration of miR-21 in the diluted sample is calculated to be 88 pM while the undiluted sample was measured to be 185 pM, showing a nearly linear response to dilution, as expected.

(iii) Effect of non-target miRNA on the IEM sensor

The selectivity of the IEM sensor is the key factor in its reliability for miRNA quantification. However, human plasma contains various miRNA species that might provide non-specific hybridization and which in turn would show some signal bias. To understand the specificity of the IEM sensor to the target miRNA miR-21, an IEM sensor functionalized with the miR-21 probe

was tested against a non-target miRNA by spiking 1 μM of miR-196a into a 20 μL healthy human plasma sample. At this high concentration, any non-specific binding due to miR-196a should be readily measurable. Furthermore, miR-196a already exists in blood plasma and is known to serve as a biomarker for cancer[5], and hence represents a potential non-specific miRNA that could bias the IEM sensor in clinical samples via non-specific binding. Here we did not lyse the sample so that we could determine if the spiked free-floating miR-196a non-selectively binds to the IEM sensor. As shown in Supplementary Fig. 7, both the control and miR-196a spiked plasma samples produced the same voltage shift of approximately 0.17 V, which is the value due to the free-floating miR-21 alone. This confirms that non-specific binding does not affect the IEM sensor measurements.

(iv) IEM response when functionalized with a man-made synthetic probe

To test if any molecules present in the plasma sample non-specifically bind to a probe on the IEM sensor, a man-made synthetic sequence (sequence GATCGCAGCCAAATGACGTGAC purchased from Integrated DNA Technologies), not complimentary to any known nucleic acid, was used as a probe and functionalized to an IEM. Supplementary Fig. 8 shows the measured voltage shift both before and after SAW lysing. The measured voltage shifts for both cases in on the order of the noise level in the CVC measurements, indicating that no plasma species attached to the synthetic probe.

(v) Confirming the detection of free-floating miRNA

To confirm that free-floating miRNA are in fact detected by the integrated device, a control experiment was conducted where RNase was added to a non-lysed plasma sample to breakdown any free-floating RNA. 1 μL of RNase A (Qiagen) was added to 100 μL of healthy human plasma sample and incubated at 65˚C for 10 minutes to annihilate the free-floating RNA. Supplementary Fig. 9 shows the integrated device results for both untreated plasma and plasma mixed with RNase. After digesting the free-floating miRNAs using RNase, a small voltage shift, less than the limit of detection, was measured by the integrated device but a typical voltage shift was measured for

untreated plasma. This confirms that the integrated device does in fact measure free-floating miRNA as indicated in Fig. 3 of the main manuscript.

(vi) Eliminating negatively-charged proteins as a potential interferent

Lysing EVs in plasma using the SAW device will result in the release of many different biomolecules, including negatively-charged proteins, that might interfere with target miRNA detection by remaining associated with hybridized target miRNAs. Such interference could affect the resulting CVC characteristics. In order to prove the specificity of the functionalized IEM sensor towards the target miRNA, a SAW-lysed plasma sample was treated with protease to break down any proteins before introducing the sample to the concentration/sensing chip. 2 μL of Proteinase K (Qiagen) was added to 20 μL of SAW-lysed healthy human plasma sample. Supplementary Fig. 10 shows that the voltage shifts of protease-treated and untreated SAW-lysed plasma samples are almost equal, indicating that associated proteins do not affect the measurement.

Comparison of Lysing Efficiency between the SAW Lysing Chip and a Commercial Kit

In order to examine the relative performance of SAW-based EV lysing to conventional approaches, we compared it to a commercially available lysing and miRNA extraction kit (see Methods). First, 50 μL of healthy human plasma was passed through the SAW lysis chip followed by miRNA extraction using a commercial ExoQuick™ Plasma Prep kit (System Biosciences Inc.) and RT-qPCR analysis. For comparison, a second 50 μL plasma sample that did not undergo SAW lysis was also analyzed using the same commercial kit followed by RT-qPCR. The TRIzol®-based lysis step is included in the miRNA extraction protocol, such that the SAW-lysed sample was first lysed by SAW and then by TRIzol® while the other sample was only lysed by TRIzol®. The measured RT-PCR miR-21 expression levels normalized by the spiked-in miR-39 are shown in Supplementary Fig. 11. The miR-21 concentration was measured to be 50% greater for the SAW-lysed plasma sample, which shows that SAW-based lysing is more efficient than TRIzol®-based lysing.

As an additional control, a healthy human plasma sample was chemically lysed using the same TRIzol® method instead of SAW-based lysing. 28 µL of purified lysed sample was obtained utilizing 200 µL of plasma suggesting a 7.142× dilution, which has been taken into account in the calculation. The chemically-lysed plasma sample was then introduced into the concentration/sensing chip and the voltage shift was measured. Supplementary Fig. 12a shows the voltage shifts for plasma and SAW-lysed plasma, and Supplementary Fig. 12b shows the voltage shift for the chemically-lysed plasma, both indicating the presence of miR-21 in the sample. When converted to concentration, the SAW device produced 55 pM of mir-21 and the chemical lysate produced 30 pM, 46% less than the SAW device confirming the effectiveness of SAW for EV lysing.

Standard Curve for RT-PCR of miR-21 and miR-39

The standard curves for RT-PCR were generated using a series of dilutions of synthetic miR-21 and miR-39 and are shown in Supplementary Fig. 13. The reverse transcription and qPCR were conducted using the protocols described in the Methods section. The curves show good amplification efficiency and linear behavior for both miRNAs across 4 orders of magnitudes.

Supplementary References

[1] Valadi, H., Ekström, K., Bossios, A., Sjöstrand, M., Lee, J. J. & Lötvall, J. O. Exosome-mediated transfer of mRNAs and microRNAs is a novel mechanism of genetic exchange between cells. *Nat. Cell. Biol.* **9,** 654 (2007).

[2] Mathivanan, S., Ji, H. & Simpson, R. J. Exosomes: extracellular organelles important in intercellular communication. *J. Proteom.* **73,** 1907-1920 (2010).

[3] Théry, C. et al. Minimal information for studies of extracellular vesicles 2018 (MISEV2018): a position statement of the International Society for Extracellular Vesicles and update of the MISEV2014 guidelines. *J. Extracell. Vesicles*. **7**, 1535750 (2018).

[4] Lu, H., Schmidt, M. A. & Jensen, K. F. A microfluidic electroporation device for cell lysis. *Lab Chip* **5,** 23-29 (2005).

[5] Hezova, R. et all. Evaluation of SNPs in miR-196-a2, miR-27a and miR-146a as risk factors of colorectal cancer. *World J. Gastroenterol* **18,** 2827 (2012).
