# Supplementary material for: Extracellular vesicle microRNA quantification from plasma using an integrated microfluidic device
Source: Commun Biol. 2019 May 20;2:189. doi: 10.1038/s42003-019-0435-1 (PMC6527557; doi:10.1038/s42003-019-0435-1)
Supplement: Supplementary file 2 — Description of Supplementary Data [file 42003_2019_435_MOESM2_ESM.pdf]

Description of Additional Supplementary Files

File Name: Supplementary Data 1
Description: Excel file with data for all figures in main text.
