# Supplementary material for: Extracellular vesicle microRNA quantification from plasma using an integrated microfluidic device
Source: Commun Biol. 2019 May 20;2:189. doi: 10.1038/s42003-019-0435-1 (PMC6527557; doi:10.1038/s42003-019-0435-1)
Supplement: Supplementary file 3 — Reporting Summary [file 42003_2019_435_MOESM3_ESM.pdf]

nature research

Corresponding author(s): David Go

Last updated by author(s): Apr 16, 2019

Reporting Summary

Nature Research wishes to improve the reproducibility of the work that we publish. This form provides structure for consistency and transparency in reporting. For further information on Nature Research policies, see Authors & Referees and the Editorial Policy Checklist.

Statistics

For all statistical analyses, confirm that the following items are present in the figure legend, table legend, main text, or Methods section.

n/a	Confirmed	
☐	☒	The exact sample size (*n*) for each experimental group/condition, given as a discrete number and unit of measurement
☐	☒	A statement on whether measurements were taken from distinct samples or whether the same sample was measured repeatedly
☐	☒	The statistical test(s) used AND whether they are one- or two-sided<br>*Only common tests should be described solely by name; describe more complex techniques in the Methods section.*
☒	☐	A description of all covariates tested
☒	☐	A description of any assumptions or corrections, such as tests of normality and adjustment for multiple comparisons
☐	☒	A full description of the statistical parameters including central tendency (e.g. means) or other basic estimates (e.g. regression coefficient) AND variation (e.g. standard deviation) or associated estimates of uncertainty (e.g. confidence intervals)
☒	☐	For null hypothesis testing, the test statistic (e.g. *F*, *t*, *r*) with confidence intervals, effect sizes, degrees of freedom and *P* value noted<br>*Give P values as exact values whenever suitable.*
☒	☐	For Bayesian analysis, information on the choice of priors and Markov chain Monte Carlo settings
☒	☐	For hierarchical and complex designs, identification of the appropriate level for tests and full reporting of outcomes
☒	☐	Estimates of effect sizes (e.g. Cohen's *d*, Pearson's *r*), indicating how they were calculated

Our web collection on statistics for biologists contains articles on many of the points above.

Software and code

Policy information about availability of computer code

Data collection	No software was used.
Data analysis	OriginPro - but the statistics were also checked independently by hand.

For manuscripts utilizing custom algorithms or software that are central to the research but not yet described in published literature, software must be made available to editors/reviewers. We strongly encourage code deposition in a community repository (e.g. GitHub). See the Nature Research guidelines for submitting code & software for further information.

Data

Policy information about availability of data

All manuscripts must include a data availability statement. This statement should provide the following information, where applicable:

- Accession codes, unique identifiers, or web links for publicly available datasets
- A list of figures that have associated raw data
- A description of any restrictions on data availability

The authors declare that the main data supporting the findings of this study are available within the article, and data sets for the main figures are in its Supplementary Information files. Additional data, such as for Supporting Figures, are available from the corresponding author DBG upon request.

Field-specific reporting

Please select the one below that is the best fit for your research. If you are not sure, read the appropriate sections before making your selection.

☒ Life sciences ☐ Behavioural & social sciences ☐ Ecological, evolutionary & environmental sciences

Life sciences study design

All studies must disclose on these points even when the disclosure is negative.

Sample size	We used six healthy human plasma donor samples, three healthy and three liver cancer mice samples (see below), and three human liver cancer clinical samples for this study. This sample size was sufficient to obtain acceptable error bars and draw a conclusion about the analytical performance of the integrated microfluidic device.
Data exclusions	No data were excluded from the integrated microfluidic device study. For the comparison to RT-qPCR analysis, Ct values greater than negative control were excluded from the final analysis.
Replication	Considering all the measurements, a standard deviation and uncertainties were calculated as described in the manuscript. All attempts at replication were successful.
Randomization	We used de-indentified healthy human plasma samples as purchased from the vendor, ZenBio, and de-identified human liver cancer samples were obtained under an approved IRB (for B.L.S.) from the University of Southern California tissue bank
Blinding	In terms of blinding the study, different unknown concentrations of synthetic target samples were run in triplicate using the integrated microfluidic device, and the results were found to be in accordance with the calibration plot obtained using known target concentrations, as shown in Figure 2b in the manuscript.

Reporting for specific materials, systems and methods

We require information from authors about some types of materials, experimental systems and methods used in many studies. Here, indicate whether each material, system or method listed is relevant to your study. If you are not sure if a list item applies to your research, read the appropriate section before selecting a response.

Materials & experimental systems

n/a	Involved in the study
☒	☐ Antibodies
☒	☐ Eukaryotic cell lines
☒	☐ Palaeontology
☐	☒ Animals and other organisms
☒	☐ Human research participants
☒	☐ Clinical data

Methods

n/a	Involved in the study
☒	☐ ChIP-seq
☒	☐ Flow cytometry
☒	☐ MRI-based neuroimaging

Animals and other organisms

Policy information about studies involving animals; ARRIVE guidelines recommended for reporting animal research

Laboratory animals	Genetically Modified Male C57/Bl6 mice at 15 months of age were used for this study.
Wild animals	N/A
Field-collected samples	N/A
Ethics oversight	For the mouse samples, all procedures and protocol were reviewed and approved by the University of Southern California Institution Animal Care and Use Committee.

Note that full information on the approval of the study protocol must also be provided in the manuscript.
